# Precursor T-Lymphoblastic Lymphoma Presenting With a Cough: A Case Report

**DOI:** 10.7759/cureus.25011

**Published:** 2022-05-15

**Authors:** Anmol Taneja, Samarth Shukla, Sourya Acharya, Sunita Vagha

**Affiliations:** 1 Department of Pathology, Jawaharlal Nehru Medical College, Datta Meghe Institute of Medical Sciences (Deemed to be University), Wardha, IND; 2 Department of Medicine, Jawaharlal Nehru Medical College, Datta Meghe Institute of Medical Sciences (Deemed to be University), Wardha, IND

**Keywords:** histopathology, ct pulmonary angiogram, t-lymphoblastic lymphoma, non hodgkin’s lymphoma, lymphadenopathy, productive cough

## Abstract

Lymphoblastic lymphoma (LBL) is a rare subtype of non-Hodgkin lymphoma (NHL) and the majority (85-90%) of the cases are comprised of precursor T-lymphoblastic lymphoma (T-LBL). We report a case of a 17-year-old male who presented with a productive cough for one month along with complaints of difficulty in breathing (unrelated to exertion) for four days and chest pain (left-sided, non-radiating) for two days. On clinical examination, lymphadenopathy was observed; mid jugular cervical lymph nodes were palpable on the left side, which were non-tender, matted, and approximately 1 x 1 cm in diameter. CT pulmonary angiogram showed a diffuse isodense mass in the mediastinum involving perivascular, pretracheal, paratracheal, and subcranial spaces. CT findings suggested multiple lymph nodal masses, possibly lymphoma. On histopathology, it was initially reported as NHL and, on immunohistochemistry, it was confirmed as T-LBL. A thorough clinical examination of the patient along with appropriate investigations is required to reach a precise diagnosis and achieve favorable outcomes. This case is unique as the patient presented with a cough and was reported to have NHL on histopathology.

## Introduction

An aggressive neoplasm produced from T-cell progenitors accounts for up to 15 and 25% of all acute lymphocytic lymphoma (ALL) cases in children and adults, respectively [1]. Precursor T-lymphoblastic lymphoma (T-LBL) accounts for about 85-90% of all lymphoblastic lymphoma (LBL) cases [2]. The condition has a typical clinical presentation. Most of the cases involve individuals of young age groups and present with a mediastinal mass as well as enlargement of lymph nodes [3]. Untreated patients present with a clinical course that is highly aggressive, with rapid multisystem involvement, leukemic blood picture, and even death after a few months [4]. Modalities such as biopsy, histopathology, fine needle aspiration cytology (FNAC), and immunohistochemistry (IHC) are the mainstays of diagnosis [2]. Patients with T-ALL/LBL have reportedly had a dramatic improvement in their outcomes thanks to more effective and precise treatment methods, and five-year survival rates in this patient population have risen to over 80% in children and over 50% in adults [1].

## Case presentation

A 17-year-old-male child presented to the medicine OPD with complaints of productive cough for one month, difficulty in breathing (unrelated to exertion) for four days, and chest pain (left-sided, non-radiating) for two days. On clinical examination, lymphadenopathy was present; mid jugular cervical lymph nodes were palpable on the left side, which were noted to be non-tender, matted, and approximately 1 x 1 cm in diameter, while the other regional lymph nodes were not palpable. On cardiovascular examination, an ejection systolic murmur over the pulmonary area was found. Respiratory examination revealed a tracheal shift to the right side with reduced entry to the right side.

**Figure 1:**
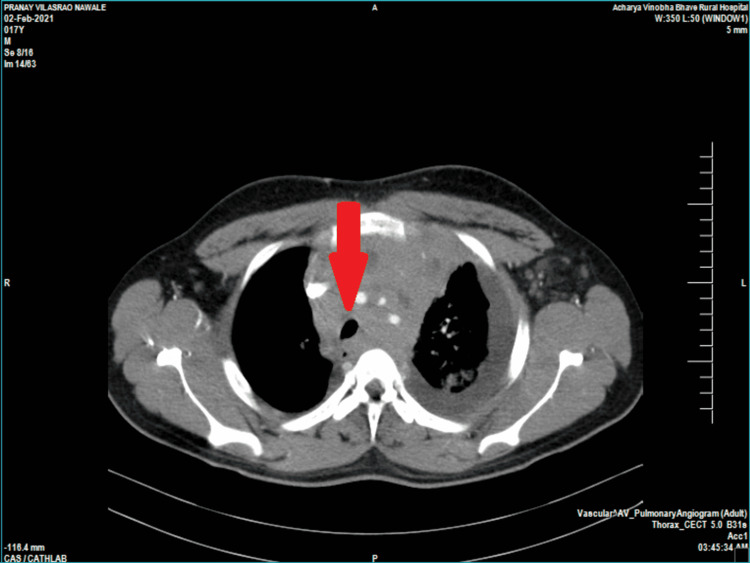
CT depicting isodense mass in the paratracheal region (arrow) CT: computed tomography

Peripheral smear suggested neutrophilic leukocytosis and otherwise normal morphology of red blood cells with adequate platelets. Pleural fluid examination revealed exudative effusion, whereas en-bloc cytology of pleural fluid was suggestive of marked lymphoid cell infiltrates with atypia. CT pulmonary angiogram showed a diffuse isodense mass in the mediastinum involving perivascular, pretracheal, paratracheal, and subcranial spaces, as shown in Figure 1. CT findings were suggestive of multiple lymph nodal masses, possibly lymphoma.

**Figure 2:**
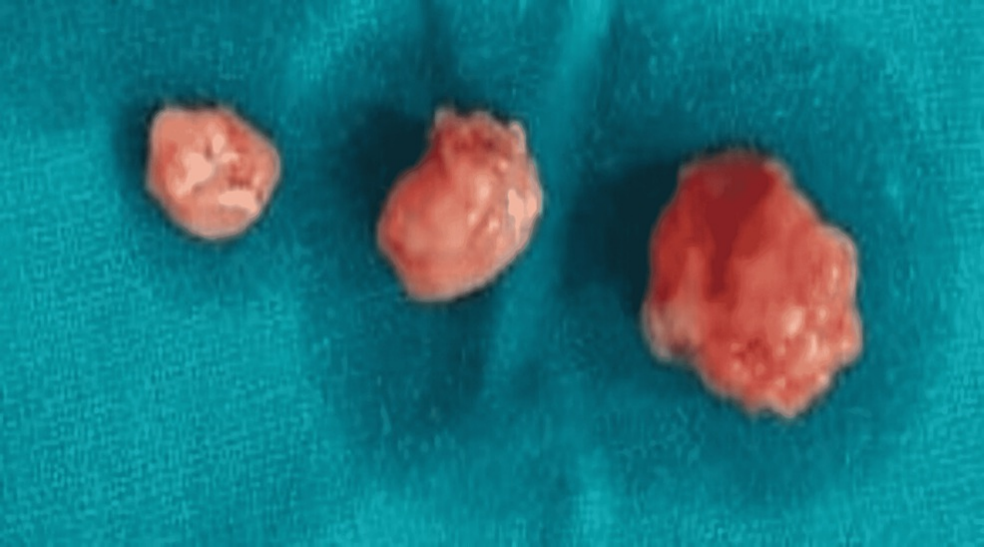
Gross appearance: three brownish, irregular lymph nodes

FNAC of the left jugular lymph node was suggestive of atypical lymphoid hyperplasia. Lymph nodes were excised and sent for a histopathological examination. Grossly, three lymph nodes were identified, aggregating approximately 1.5 x 1.5 x 0.5 cm, as depicted in Figure 2.

**Figure 3:**
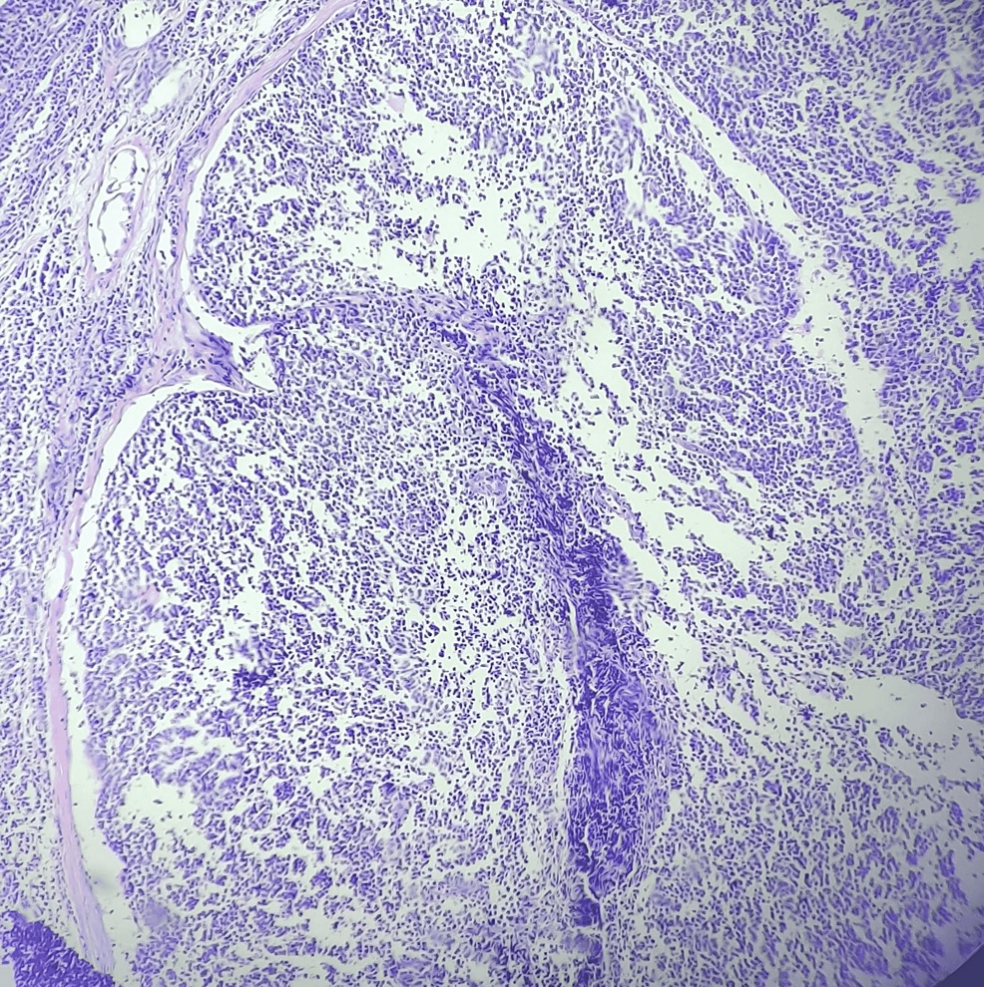
Microscopy H & E staining of 10x magnification The figure shows an intact lymph node with a capsule; the architecture of the nodes appeared to be effaced

**Figure 4:**
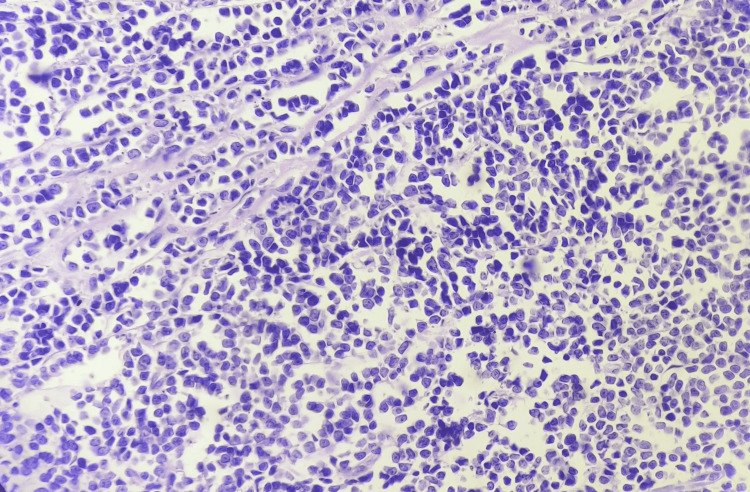
Microscopy H & E staining of 40x magnification

Microscopically, the section from the left supraclavicular lymph node area showed an intact lymph node with a capsule, and the architecture of the nodes appeared to be effaced (Figures 3, 4). The histopathological features raised suspicion for NHL.

**Figure 5:**
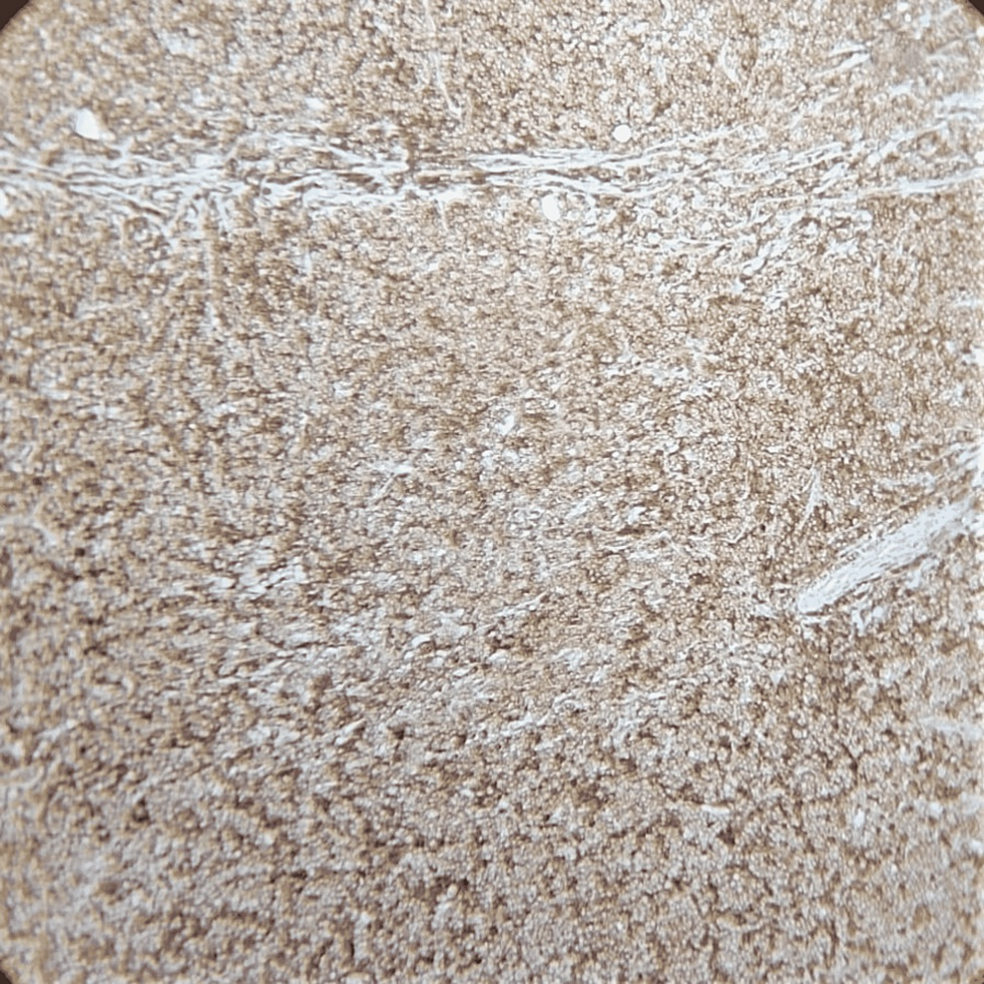
Diffuse and strong immunoreactivity for CD99, CD4, and CD3

**Figure 6:**
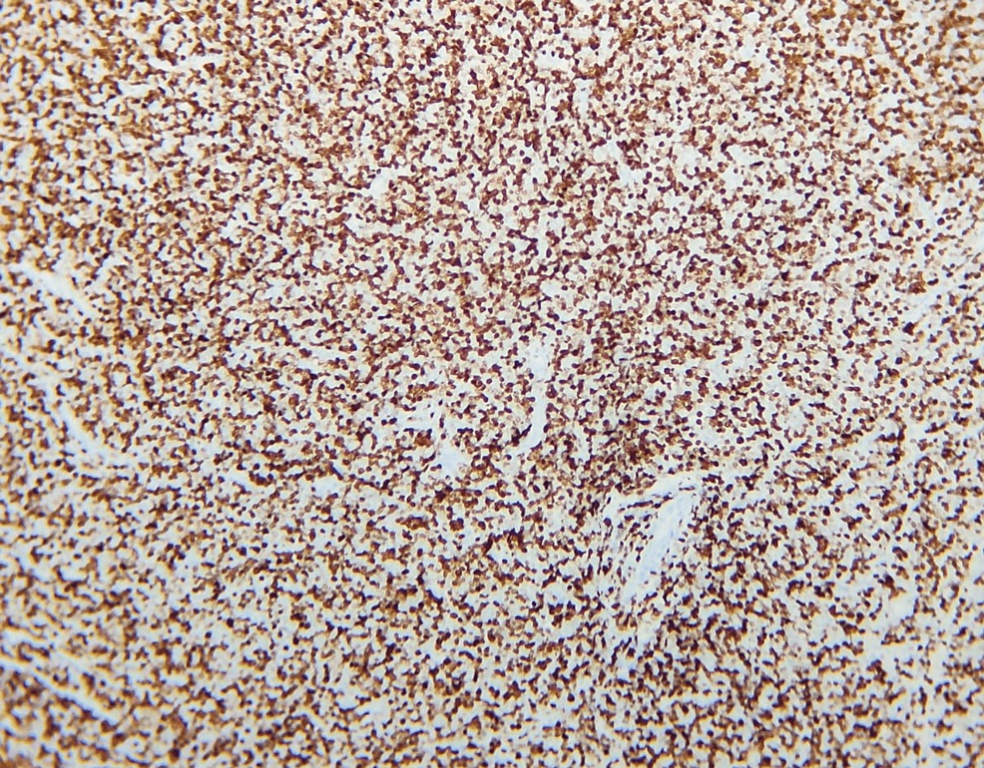
MIB labeling index of 80%

**Figure 7:**
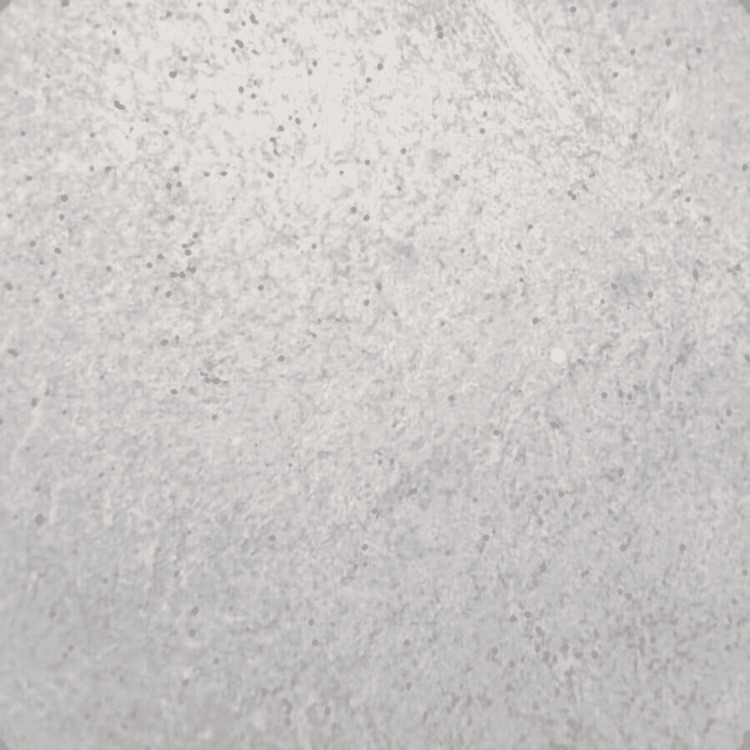
Immunonegativity for CD20, CD79a, and CD8

IHC for the confirmation of the diagnosis was advised, and it showed neoplastic lymphoid cells showing diffuse and strong immunoreactivity for TdT, CD99, CD3, and CD4 while immunonegative for CD8 and CD20. MIB-1 labeling index was 95%. The final report documented the case as T-LBL, as shown in Figures 5-7.

The patient was treated according to the modified Berlin-Frankfurt-Munster (BFM-90) protocol under the induction and maintenance phase, and the drugs used were prednisone, vincristine, daunomycin, asparaginase, methotrexate, 6-mercaptopurine, cyclophosphamide, and cytosine arabinoside. Currently, the patient is asymptomatic.

## Discussion

Lymphoma is the third most prevalent neoplasm in children and adolescents. The four subtypes of NHL in children include lymphocytic lymphoma, Burkitt lymphoma, diffuse large B-cell lymphoma (DLBCL), and anaplastic large cell lymphoma [5]. Mediastinal mass is in the thymic region in 50% of cases [4]. Lymphocytic lymphoma has a proclivity for spreading to the bone marrow or the central nervous system (CNS), and it is classified as a stage III/IV malignancy [5].

Cai et al. have reported a case of a girl child who was diagnosed with primary DLBCL in the retroperitoneal as well as gastrointestinal regions at the same time. Renal colic was the initial indication of this condition, which was unusual [6]. Remnants of the thymus are often found in the mediastinal mass, and this may lead to a mistaken diagnosis of thymoma. The other differentials include Ewing sarcoma/primitive neuroectodermal tumor (PNET), Burkitt lymphoma, and mantle cell lymphoma (blastoid variant) [4].

Although CT is the preferred diagnostic method, MRI provides better soft-tissue contrast than CT [6]; however, IHC is the modality used for making the specific diagnosis [4]. T-cell markers are expressed in 80-85% of LBLs, and TdT, a marker for precursor lymphoid cells, is an immunohistochemical hallmark of LBL. Pan-T-antigens, such as CD1, CD2, CD7, CD3, and CD43, are expressed in almost all these malignancies [4]. T-LBL is usually treated with an induction chemotherapy regimen followed by consecutive reinduction and maintenance chemotherapy [7], and it is followed by protocols devised by the BFM group: BFM-90 and BFM-95.

According to this, patients with LBL stage III and IV receive induction protocol I, followed by protocol M, reintensification protocol II, and maintenance for a total therapy term of two years. In patients who relapse after disease progression, the second-line treatment is based on ALL Relapse-BFM methods. These strategies involve five- to six-day courses and include drugs like dexamethasone, vincristine, methotrexate, L-asparaginase, and intrathecal triple therapy with prednisone, cytarabine, etc. [8].

Patients with T-ALL/LBL have shown considerable improvements in their outcomes, thanks to more effective and precise chemotherapy regimens. Over 90% of T-ALL/LBL patients achieve complete remission initially, while a considerable percentage of people will develop recurrent illness later.

We reported a unique incidence of NHL, specifically LBL, in which the first symptom was a productive cough.

## Conclusions

Establishing a diagnosis in cases of lymphoma or its variants by light microscopy alone can sometimes be problematic, especially in cases where the only presenting complaint is cough; there is currently no test that can accurately identify these cancers. Hence, we should use a variety of different diagnostic modalities, such as IHC, along with patients' detailed history and clinical examination and other necessary investigations like radiology, to establish the specific diagnosis. The accuracy rate of IHC has been reported to be quite satisfactory. Thus, IHC can be helpful in narrowing down the differential diagnosis of the NHL and its variants. This enables the patient to receive treatment at an early stage, resulting in better outcomes, prognosis, and survival rates.
